# Highly tumorigenic hepatocellular carcinoma cell line with cancer stem cell-like properties

**DOI:** 10.1371/journal.pone.0171215

**Published:** 2017-02-02

**Authors:** Benoit Lacoste, Valérie-Ann Raymond, Shamir Cassim, Pascal Lapierre, Marc Bilodeau

**Affiliations:** 1 Laboratoire d’hépatologie cellulaire, Centre de recherche du Centre hospitalier de l'Université de Montréal (CRCHUM), Montréal, Québec, Canada; 2 Département de médecine, Université de Montréal, Montréal, Québec, Canada; University of Navarra School of Medicine and Center for Applied Medical Research (CIMA), SPAIN

## Abstract

There are limited numbers of models to study hepatocellular carcinoma (HCC) *in vivo* in immunocompetent hosts. In an effort to develop a cell line with improved tumorigenicity, we derived a new cell line from Hepa1-6 cells through an *in vivo* passage in C57BL/6 mice. The resulting Dt81Hepa1-6 cell line showed enhanced tumorigenicity compared to Hepa1-6 with more frequent (28±12 vs. 0±0 lesions at 21 days) and more rapid tumor development (21 (100%) vs. 70 days (10%)) in C57BL/6 mice. The minimal Dt81Hepa1-6 cell number required to obtain visible tumors was 100,000 cells. The Dt81Hepa1-6 cell line showed high hepatotropism with subcutaneous injection leading to liver tumors without development of tumors in lungs or spleen. *In vitro*, Dt81Hepa1-6 cells showed increased anchorage-independent growth (34.7±6.8 vs. 12.3±3.3 colonies; *P*<0.05) and increased *EpCAM* (8.7±1.1 folds; *P*<0.01) and β*-catenin* (5.4±1.0 folds; *P*<0.01) expression. A significant proportion of Dt81Hepa1-6 cells expressed EpCAM compared to Hepa1-6 (34.8±1.1% vs 0.9±0.13%; *P*<0.001). Enriched EpCAM^+^ Dt81Hepa1-6 cells led to higher tumor load than EpCAM^-^ Dt81Hepa1-6 cells (1093±74 vs 473±100 tumors; *P*<0.01). The *in vivo* selected Dt81Hepa1-6 cell line shows high liver specificity and increased tumorigenicity compared to Hepa1-6 cells. These properties are associated with increased expression of EpCAM and β-catenin confirming that EpCAM^+^ HCC cells comprise a subset with characteristics of tumor-initiating cells with stem/progenitor cell features. The Dt81Hepa1-6 cell line with its cancer stem cell-like properties will be a useful tool for the study of hepatocellular carcinoma *in vivo*.

## Introduction

Hepatocellular carcinoma (HCC) is the fifth most frequent cancer and the third most deadly cancer worldwide [1] Overall survival rates are typically less than 5% [2]. It is rarely diagnosed early enough to perform curative therapy which partly explains its poor prognosis [3]. Over the years, several animal models have been developed to study its pathogenesis and evaluate potential therapies. These models are categorized as either chemically-induced, genetically-modified or xenograft models [4]. Cell lines are widely used to study HCC *in vitro* but they do not systematically give rise to solid tumors when implanted *in vivo* [5]. This has led to the frequent use of immunodeficient animals as hosts despite the difficult translation of data gained with these models to human HCC.

The main paradigm underlying cancer development over the last 40 years has been the clonal selection model in which cell clones with the highest tumorigenicity are at the origin of the tumor mass [6]. On the other hand, the cancer stem cells/tumor initiating cells (TIC) theory, which has emerged in the last decade, suggests that cancer cells are divided in subpopulations with different characteristics, such as the ability to form new tumors, resist chemotherapy or divide rapidly [7]. Increased tumorigenicity can therefore occur either by increasing the subpopulation of TIC in the cell pool and/or by selecting a cell lineage that has developed a particular ability to grow in a defined environment.

TIC are known to express a number of characteristic cell surface markers which facilitates their identification [8, 9]. Among these markers, the epithelial cell adhesion molecule (EpCAM), a type 1 transmembrane glycoprotein, is exclusively expressed in epithelial-derived cells [10]. Its down regulation by siRNA in gastric cancer cell lines is accompanied by a lower clonal colony rate, anchorage-independent growth and *in vivo* tumorigenicity [11]. In the Huh-7 hepatoma cell line, selection of EpCAM-positive cells has been associated with enhancement of *in vivo* tumorigenicity and *in vitro* characteristics associated with aggressiveness, such as anchorage independent growth [12]. Inhibition of EpCAM by siRNA has been associated with loss of tumorigenicity in a murine model of HCC [13].

Currently, most HCC murine models require immunodeficient animals thereby limiting the translation of data gained in these models to human HCC. Natural selection of tumorigenic cells under constant surveillance by the immune system is a key aspect of cancer development [14]. The Hepa1-6 clone, which was isolated from the BW7756 tumor that arose spontaneously in the C57L/J mouse strain, is widely used, well characterized *in vitro* and shows high expression of alpha-fetoprotein (AFP) [15]. Herein, we performed an *in vivo* passage of Hepa1-6 cells in C57BL/6 mice in an effort to isolate HCC cells with tumor-initiating and stem/progenitor cell features. Seventy days after intrasplenic (IS) inoculation, a solid liver tumor was observed in one mouse. Cells isolated from this tumor showed a different morphology than Hepa1-6 cells *in vitro*. The newly derived cell line was designated Dt81Hepa1-6 and subsequently characterized *in vivo* and *in vitro*. Herein, we show that *in vivo* passage has led to a hepatocellular carcinoma cell line with enhanced tumorigenicity and EpCAM expression, hallmark characteristics of tumor initiating cells (TIC).

## Materials and methods

### Reagents

Soft agar, Bacto-agar powder and Type 1 collagen (COL1) were purchased from BD Biosciences (Mississauga, On, Canada). TRIZOL reagent was purchased from Invitrogen (Burlington, On, Canada). Quantitect reverse transcription kit, Taq DNA polymerase kit and SYBRGreen kit were purchased from QIAGEN (Toronto, On, Canada). Developer and fixation solution kits were purchased from Kodak (Rochester, NY, USA). Unless stated otherwise, all other products were from Sigma-Aldrich (Oakville, On, Canada).

### Animals

Male C57BL/6 mice (20g) were bought from Charles River (Saint-Constant, Qc, Canada) and fed *ad libidum* with normal chow. Animals were monitored daily for their appearance, state of hydration, behavior and clinical signs. Humane endpoints were in place during the study. General endpoints included loss of 20% or more of body weight, aggressiveness associated with pain or uncontrollable pain, prolonged anorexia, prostration, dehydration and nervous disorder. Specific endpoints for tumors were also included: Mass of the tumor increased to the extent as to interfere significantly with normal functions or induce suffering and/or distress, ulceration and/or infection at the site of the tumor, invasion of neighboring tissues by localized tumor, self-induced trauma or a tumor load exceeding 10% of body weight of the animal. No animals died prior to the experimental endpoint. Animals were sacrificed by exsanguination under anesthesia (Induction with inhaled 4% Isoflurane and maintenance with inhaled 2% Isoflurane). All procedures were performed in accordance with Canadian Council on animal care and approved by the *Comité institutionnel de protection animale (CIPA) du CHUM (Canada)*.

### Cell line and culture

Authenticated Hepa1-6 murine hepatoma cell line was bought from the American Type Culture Collection (Manassas, Virginia, USA) for this project. All cultures were maintained at 37°C and 5% CO_2_. Cell lines were cultured in Dulbecco's modified eagle's medium (DMEM) supplemented with 10% FBS. All products used to culture cells were from Invitrogen (Carlsbad, CA, USA). Dishes coated with COL1 [13.9μg/cm^2^] were prepared as previously described [16].

### Intrasplenic cell injection

The Hepa1-6 or the Dt81Hepa1-6 cell lines were trypsinized and resuspended in saline solution containing 0.25% albumin at the selected concentration. A cell aliquot (200μL) was loaded in 21G syringes for intrasplenical injection. Under anesthesia, an abdominal incision was performed and the spleen was pulled out on a 37°C saline-soaked gauze. The syringe was mixed and the needle inserted in the spleen parenchyma and cells were slowly injected. When the spleen regained its bright red color, the needle was slowly drawn back and a droplet of Vetbound veterinary glue (3M, London, On, Canada) applied. The spleen was put back into the abdominal cavity and the abdominal incision closed.

### Dt81Hepa1-6 cell line isolation

Thirty C57BL/6 mice were injected intrasplenically with 1M Hepa1-6 cells and sacrificed at regular intervals to monitor for the development of liver tumors. In one mouse sacrificed 70 days after splenic injection, a liver tumor was observed. This tumor was excised and gently sliced and incubated at 37°C in DMEM containing type IV collagenase [0,35mg/mL] (Worthington Biochemical Corp. Lakewood, NJ, USA) and CaCl_2_ [1mg/mL] for 15min under agitation. The cellular solution was then washed 3-times. The final cell suspension was seeded at a 0.25M cells/mL on COL1-coated dishes in supplemented medium. Medium was changed until cells reached confluence. Cells were then pooled and cultured in 75cm^2^ flasks.

### In vivo characterization of Dt81Hepa1-6 cells

To determine time response curve, 1M Dt81Hepa1-6 cells were injected intrasplenically in C57BL/6 mice and sacrificed 3.5, 7, 14, 21 or 28 days later. Increasing cell concentrations (1K, 10K, 0.1M, 0.5M and 1M cells) were injected intrasplenically in C57BL/6 to build a dose response curve. Animals were sacrificed 28 days after injections.

### Cell aggregation assay

The protocol was adapted from the slow aggregation protocol [17]. 96-well plates were covered with 50μL of a sterile soft agar gel [1.3%] in lactated Ringer’s solution (Baxter, Mississauga, On, Canada) to prevent cell adhesion. Medium (100μL) containing 20K single cells was added to the well and left standing for 24h. The cell suspension was set between a slide and coverslip and the number of cells per aggregate was counted using a Zeiss Axioplan 2 microscope (Göttingen, Germany) at 400x magnification. Representative microphotographs were obtained with the Northern Eclipse 6.0 software (Empix Imaging, Missisauga, On, Canada).

### Soft-agar colony formation assay

To evaluate anchorage-independent growth, individual cells were seeded in a soft agar gel [11, 18]. Briefly, 6-well plates were filled with 2mL of warm 0.6% soft agar solution and allowed to solidify. Then, 1mL of a warm soft agar 0.3% mixture containing 10K freshly trypsinized single cells was poured on top of each well, left to polymerize and then, an extra 2mL of the warm solution [0.6%] was added on top. Afterwards, cells were maintained at 37°C with 1mL of supplemented medium; medium was changed every week for 5 weeks. Visible foci were counted manually and representative microphotographs were obtained at 25x.

### Colony-forming cell assay

The ability of independent cells to form colonies without cell-to-cell contact was assessed by a proliferative clonal colony assay as previously described [11]. Briefly, each cell line was seeded at a concentration of 1000 cells per 2mL of supplemented media to obtain single attached cells and thus isolated foci of proliferation. Media was changed once every week for 4 weeks. At the end of experiment, the number of colonies per well was manually counted.

### Double-layer gel droplets cell invasion assay

The potential for invasiveness was evaluated with a derived version of a previously described double hemisphere invasion assay [19]. Briefly, concentrated COL1 ice-cold solution was diluted with DMEM and neutralized with NaOH in order to obtain a 3mg/mL COL1 gel. This gel was mixed with freshly trypsinized cells at a concentration of 4000 cells/μL. Droplets of 5μL were then plated at 3 different locations in a 3.5cm diameter dish and allowed to solidify at 37°C for 15min. A 30μL of the ice-cold COL1 gel was then poured on top of each cell-containing droplets and left to polymerize for 15min at 37°C. Warm supplemented medium was then gently added. Every 12h, for 168h, dishes were fixed and nuclei stained with Hoechst 33258 [50ng/mL]. Cells invading the outer layer were counted manually. Area under the curve (AUC) of time-dependent invasion was calculated for each experiment and used for statistical analysis. Representative microphotographs were obtained using Northern Eclipse 6.0 software under a fluorescent Zeiss Axioplan 2 microscope at 25x magnification.

### Wound Healing Assay (WHA) and COL1-Embedded WHA

In both assays, cells were seeded at 0.160M cells/cm^2^ for Hepa1-6 and 0.320M cells/cm^2^ for Dt81Hepa1-6 to reach near-full confluence. Media was changed after 24h or when cells reached full confluence. Media was removed, and using a 200μL pipette tip, a line was scratched at 3 different locations changing tip each time. Dishes were then rinsed 3 times with warm PBS to remove floating cells. Fresh supplemented medium was added. COL1-embedded WHA experimental dishes were covered with a layer of 2 mL COL1-[3mg/mL]-supplemented medium and covered with fresh supplemented medium polymerization of the gel at 37°C for 15min. Microphotographs were obtained at the beginning of the experiment and after 24h. The distance travelled by cells was measured using imageJ 1.46 software (NIH, USA).

### Cell-spreading assay

To ensure proper comparison between experiments, both cell lines were seeded at a 0.3M cells/mL in DMEM on COL1 plates. Six hours after seeding, cells were examined though an inverted microscope (Motic, Richmond, BC, Canada) and cells showing an elongated morphology were counted in 10 different fields per well at a 400x magnification.

### MTT (3-(4.5-Dimethylthiazol-2-yl)-2,5-diphenyltetrazolium bromide) viability assay

Cells were seeded at 0.125M cells/cm^2^ for Hepa1-6 and 0.250M cells/cm^2^ for Dt81Hepa1-6 and cultured overnight. Media was then replaced for fresh media with *cis*-Diammineplatinum(II) dichloride (CP) [25μg/mL] for 24h without FBS. Media was removed and 1mL of MTT solution [2mg/mL] in DMEM was added and incubated for 3h. Media was removed and dimethylsulfoxide (1mL) was added and plates gently shaken at room temperature for an hour. Plates were then read using a Synergy HT-spectrofluorometer plate reader at 495nm wavelength. Viability was expressed in percent of untreated controls.

### Cell doubling time

Cells were seeded at 0.09M cells/cm^2^ for Hepa1-6 and 0.175M cells/cm^2^ for Dt81Hepa1-6 in supplemented DMEM for 8h and the medium changed for serum-free DMEM for overnight culture. Media was changed with fresh supplemented DMEM (time 0). Cell concentration was evaluated every 24h for 72h. At each time point, cells were fixed, stained with crystal violet [0.05% w/v in 200mM MOPS buffer, pH6.0] and read using a Synergy HT plate reader at 570nm wavelength. Cell doubling time was evaluated using a last square fitting exponential method (Roth V. 2006, http://www.doubling-time.com/compute.php).

### Flow cytometry and cell sorting

For flow cytometry analysis, 1 M cells were washed, resuspended in phosphate-buffered saline (PBS) containing 0.2% BSA (FACS buffer), and incubated with APC-labeled anti-CD326 (EpCAM) antibody (Ebioscience, San Diego, California, USA) for 30 minutes at 4°C. Cells were then washed and resuspended in 200 μl FACS buffer for analysis on a FACS BD LSRII flow cytometer with DIVA6 software (both from BD Biosciences, Mississauga, On, Canada) and analyzed with FlowJo software (Tree Star, Oregon, USA). For cell sorting, forty million freshly trypsinized Dt81Hepa1-6 cells were stained with AP-labeled anti-CD326 as described above. The cells were resuspended in 20mL PBS/5% FBS/25mM HEPES and passed through a 100μm filter to avoid aggregates. Samples were sorted in DMEM/50%FCS on a FACS ARIA II (BD Biosciences, Mississauga, On, Canada).

### Histology

Formalin-fixed liver, spleen and lung samples obtained at the time of sacrifice were set in paraffin block, sliced and stained with HPS. Representative microphotographs were obtained with a Carl-Zeiss Axioplan 2 microscope at 100x magnification using the Northern Eclipse 6.0 software.

### Western blotting

Cells were seeded in supplemented media at a determined density for Hepa1-6 (0.125M cells/cm^2^) and Dt81Hepa1-6 (0.250M cells/cm^2^) for 7h on 10cm dishes coated with COL1. Media was then changed overnight for serum-free DMEM. Cells were then harvested and subjected to a 12% SDS-polyacrylamide gel electrophoresis and blotted as described [20]. Membranes were blocked and probed with the following antibodies: anti-beta-catenin, anti-ERK1/2 and anti-phosphospecific ERK1/2, anti-AKT and anti-phosphospecific AKT, anti-Bad and anti-phosphospecific Bad (all from Cell Signaling, Pickering, On, Canada), anti-Bcl-XL (Transduction Laboratories, Mississauga, On, Canada), anti-Bak (Upstate, Lake placid, NY, USA), anti-Bid (R&D system, Minneapolis, MN, USA) and anti-actin kit (Calbiochem, Merck KGaA, Darmstadt, Germany) for 2h in PBST containing 1% milk at room temperature. Membranes were washed and then incubated with HRP-conjugated secondary anti-rabbit IgG, anti-mouse IgG (both from BD Pharmingen, San Diego, California, USA), anti-rat IgA (Cedarlane labs, Burlington, On, Canada) or anti-mouse IgM (from anti-actin kit) antibodies at room temperature in PBST containing 5% milk during 1h. After extensive washes in PBST, bound peroxidase was detected with enhanced chemiluminescence blotting substrate (Perkin-Elmer, Woodbridge, On, Canada), according to the manufacturer’s instructions.

### qPCR gene expression analysis

For *in vitro* experiments, cells at 70% confluence were trypsinized, washed in L-15 media and centrifuged for 2 min at 1000rpm. The mRNA was isolated using TRIZOL according to the manufacturer guidelines. For *ex vivo* sample preparation, whole frozen liver was crushed over carbonated ice and liquid nitrogen. Three different samples of liver powder were taken for a total of 80-90mg per liver. Using a potter, samples were homogenized in 1mL TRIZOL reagent and processed according to manufacturer’s recommendations. Quantitect reverse transcription was performed on 250ng of mRNA. Albumin (Alb) expression was evaluated with Taq DNA polymerase kit for 30 cycles at a 59°C melting temperature (Tm). For other genes, mRNAs were analyzed with a RotorGene Real-time PCR and the Quantitect SybrGreen kit. For each gene tested, 35 amplification cycles at 59°C were used. The following sequences were used: *AFP* (*Afp*) (F:5’-GCTGGCCCTGGATGTGGCTC-3’; R:5’-GAGCTGGCAGCACGTGGAGG-3’), Albumin *(Alb)* (F:5’-TGCTGCGCTGAAGCCAATCCT-3’; R:5’-AAGCATGGCCGCCTTTCCAC-3’), *EpCAM* (Epcam) (F:5’-AAAGCCAAGCAGTGCAACGGCA-3’; R:5’-TGTGAACGCCTCTTGAAGCGCA-3’), β*-catenin* (*Ctnnb1*) (F:5’-GTACCTGAAGCTCAGCGCACA-3’; R:5’-AGCAGCGTCAAACTGCGTGGA-3’), *Cyclin D1 (Ccnd1*) (F:5’-TCCCTGGCTTGCTCAGTGCCTA-3’; R:5’-TGCCACACGCCATGAGACCA-3’), *Integrin-alpha1 (Itga1)* (F:5’- AATGGGCCCGTGCTCGATGA-3’; R:5’-TGTCCGGGTGTTGTACGCACT-3’) and *Integrin-*β*1 (itgb1)* (F:5’-TGCAGGTCGATCCTGTGACCCA-3’; R:5’-CCAGCAACCACGCCTGCTACAA-3’). Relative expression was evaluated using 3 reference genes: *HPRT1* (F:5’-GCTTGCTGGTGAAAAGGACCTCTCGAAG-3’; R:5’-CCCTGAAGTACTCATTATAGTCAAGGGCAT-3’, *Ppia* (F:5’-CGCGTCTCCTTCGAGCTGTTTG-3’; R:5’-TGTAAAGTCACCACCCTGGCACAT) and H2afz (F:5’-ACAGCGCAGCCATCCTGGAGTA-3’; R:5’-TTCCCGATCAGCGATTTGTGGA-3’) [21]. Relative gene expression was evaluated using the delta delta CT method [22]. Normal liver homogenates from C57BL/6 mice were used as baseline comparative controls for *AFP* mRNA expression.

### Statistical analysis

All *in vitro* data represent the values of at least three different experiments made in triplicate. All *in vivo* data represent the values of at least two different experiments using five different animals. Data are expressed as mean ± SE. Differences among groups were analyzed by Student’s *t*-test. A *P*-value below 0.05 was considered significant.

## Results

Thirty congenic C57BL/6 mice were injected intrasplenically with 1M Hepa1-6 cells and one mouse developed visible tumors seventy days later (S1 Fig). Cells from these tumors were isolated and cultured *in vitro*. These cells had a different morphology from the original Hepa1-6 cells and grew in closer aggregates than Hepa1-6 cells *in vitro* (S1D Fig). The newly derived Dt81Hepa1-6 cells had a similar level of albumin expression compared to Hepa1-6 cells and primary hepatocytes thus confirming their hepatocyte lineage (S1 Fig).

**Fig 1:**
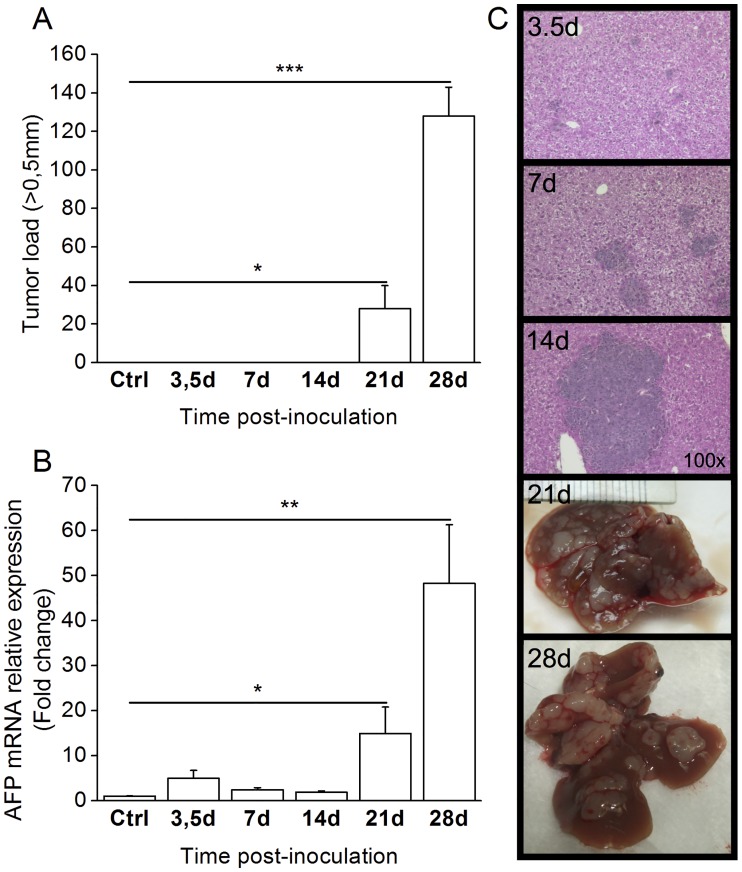
Dt81Hepa1-6 cell transfer leads to rapid tumor development. A suspension of 1M Dt81Hepa1-6 cells was injected intrasplenically in C57BL/6 mice and animals were sacrificed after 3.5 to 28 days. (A) Manual count of tumor >0.5mm. (B) Alpha-fetoprotein (AFP) (Afp) mRNA expression in liver homogenates as measured by qPCR. (C) Representative microphotographs at 100x magnification of HPS-stained liver slices obtained after 3.5, 7 and 14 days. Whole-liver photographs obtained after 21 or 28 days. Control mice were injected with 1M Hepa1-6 cells and sacrificed at each time point (3.5 to 28 days) and pooled for analysis (Ctrl). (*P<0.05; **P<0.01; ***P<0.001).

To characterize the ability of this new derived cell line to form tumors *in vivo*, we performed a time course experiment in C57BL/6 mouse. To establish its progression rate, we first inoculated 1M Dt81Hepa1-6 cells in C57BL/6 mice for a period of up to 28 days. Control mice received 1M Hepa1-6 cells and were sacrificed at each time point. Visible tumors were counted at the time of sacrifice (Fig 1A). The first macroscopic tumors (>0.5mm) appeared 21 days after intrasplenic injection in C57BL/6 mice (28±12 lesions vs control; *P*<0.05) and tumor load increased until the end of the observation period (128±15 lesions vs control; *P*<0.001). Alpha-fetoprotein expression increased significantly after 21 days (14.9±6.9 fold changes; *P*<0.05) with a further increase at 28 days (48.3±13.0 fold changes; *P*<0.01) concomitant with the appearance of tumors (Fig 1B). Microscopic tumor formation appeared 3.5 days after injection of Dt81Hepa1-6 (Fig 1C). The size of microscopic foci increased progressively between 7 and 14 days after intrasplenic (IS) injection up to the development of visible tumors (Fig 1C). In comparison, injection of Hepa1-6 in C57BL/6 resulted in only transient microscopic tumors after 21 days (S1A Fig).

**Fig 2:**
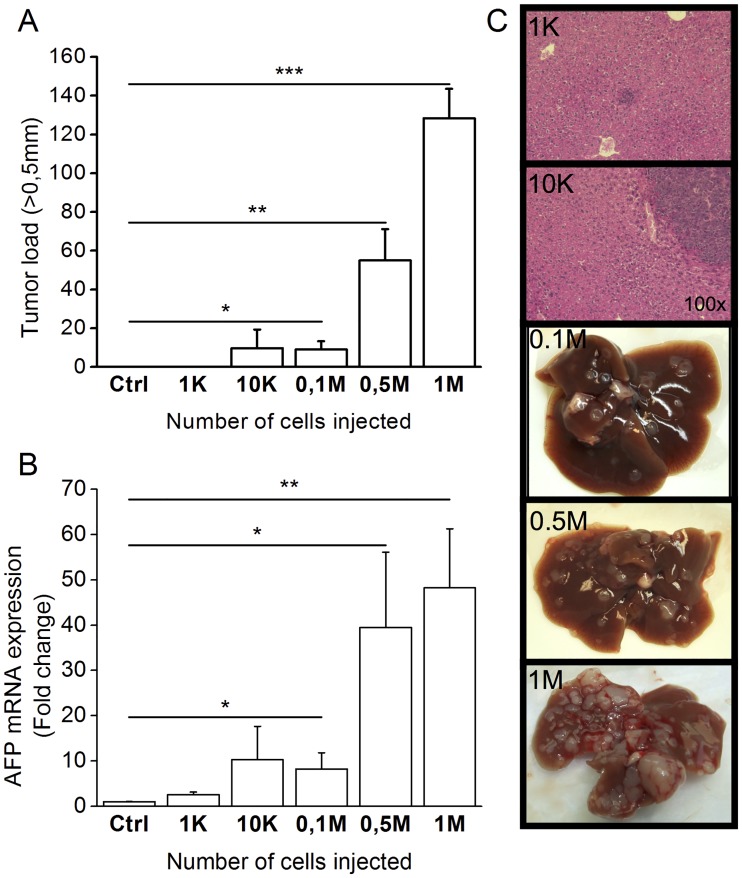
Dose-dependent tumor development following transfer of Dt81Hepa1-6 cells. Suspensions of Dt81Hepa1-6 cells [1K to 1M] were injected intrasplenically in C57BL/6 mice and animals were sacrificed after 28 days. (A) Manual count of tumor >0.5mm. (B) *AFP* (*Afp*) mRNA expression in liver homogenates by qPCR. (C) Representative microphotographs at 100x magnification of HPS-stained liver slice obtained after injection with 1K and 10K cells. Whole liver photographs of mice injected with 0.1M, 0.5M or 1M cells. Control mice were injected with 1M Hepa1-6 cells. (*P<0.05; **P<0.01; ***P<0.001).

Next, we assessed tumor development with increasing concentrations of cells injected intrasplenically in C57BL/6 mice after a 28-day period. Control mice were inoculated with 1M Hepa1-6 cells. The cell concentration threshold required to obtain visible tumors in every animal was 0.1M (9.0±5.7 lesions vs control; *P*<0.05) (Fig 2A). This was confirmed by *AFP* expression (0.1M: 8.2±3.6fold changes; *P*<0.05) (Fig 2B). Even if no visible tumors were observed, microscopic tumors could be identified with as low as 1000 and 10 000 cells (Fig 2C). Increasing cell concentration led to larger, more visible and numerous tumors (Fig 2C).

**Fig 3:**
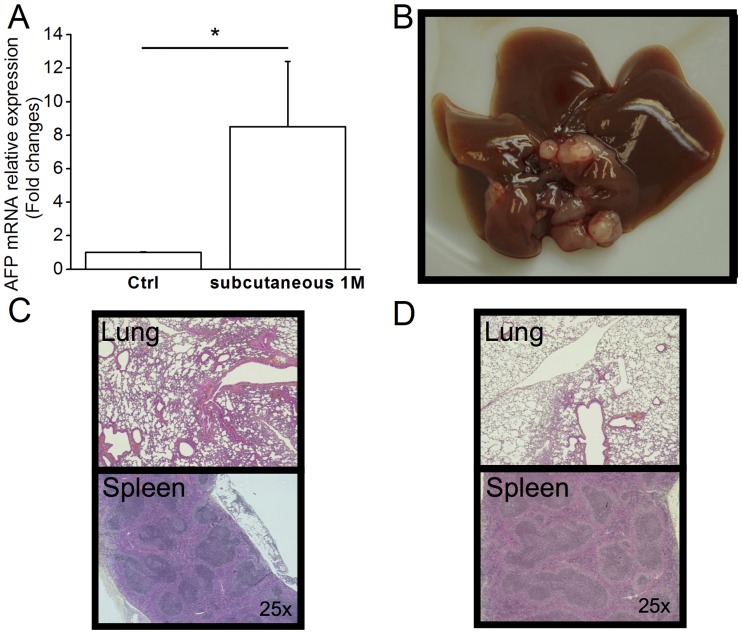
Dt81Hepa1-6 cells are specific to the liver. A suspension of 1M Dt81Hepa1-6 cells was injected subcutaneously (SC) in the left flank of C57BL/6 mice and animals were sacrificed after 28 days. (A) *AFP* mRNA relative expression in control and SC injected C57BL/6 mice. (B) Whole liver photographs at time of sacrifice. HPS-stained slices representative microphotographs of (C) lungs and spleen after SC injection and (D) Lungs and spleen after IS injection. Representative microphotographs were obtained at 100x magnification. (*P<0.05).

We assessed the specificity of implantation by Dt81Hepa1-6 cells through histological examination of the liver, spleen and lungs after intrasplenic or subcutaneous injections. Subcutaneous injection of Dt81Hepa1-6 cells in C57BL/6 mouse resulted in tumors developing macroscopically in the liver as confirmed by *AFP* expression (8.5±3.9 fold changes; *P*<0.05) but no tumor could be observed either in the lungs, subcutaneous site of injection or spleens in these mice (Fig 3). When injections were performed in the spleen, rare isolated tumor cells could be identified at the site of injection; however, no tumor cells were ever identified in the lungs of these animals (Fig 3D).

### *In vitro* neoplastic profile

**Fig 4:**
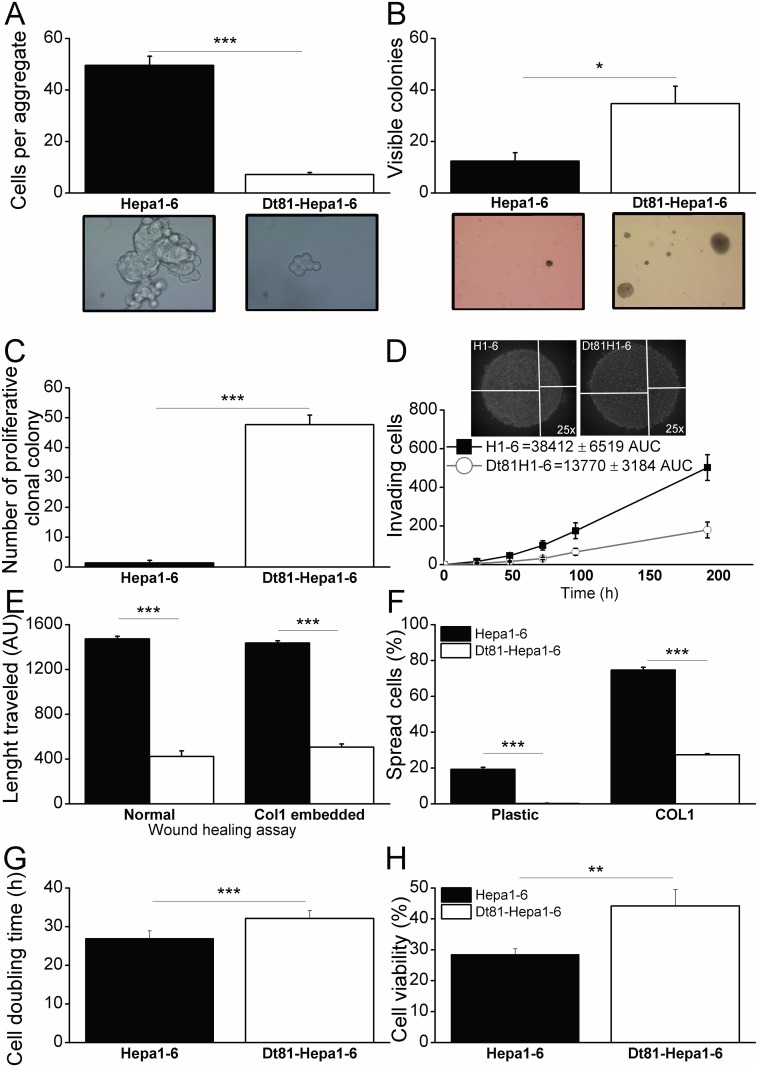
Dt81Hepa1-6 *in vitro* neoplastic profile. A cell aggregation assay was performed and (A) the number of cells per aggregate was calculated 24h after seeding both cell lines in non-adherent agar-coated wells. Representative microphotographs are shown. In order to evaluate anchorage-independent growth, a soft-agar colony formation assay was performed; (B) cell lines were seeded at 10K-cell concentration in soft-agar gel [0.3%] in order to form visible colonies and were counted 5 weeks later. Representative microphotographs of cells after 5 weeks are shown. The ability of independent cells to form colonies without cell-to-cell contact was assessed by a proliferative clonal colony assay; (C) cell lines were seeded at 3K/mL concentration to obtain isolated cells and left to grow as colonies over a 4 weeks period and counted. The potential for invasiveness was evaluated with a double-layer droplet cell invasion assay; (D) Cell lines [20K cells] were embedded in COL1 [3mg/mL] to form a double layered droplet and cells penetrating the outer COL1 layer were counted every 12h for 8 days. Area under the curves (AUC) of the time-dependent invasion was calculated and used for statistical analysis. Representative microphotographs are shown. For the wound-healing assays (WHA); (E) normal and COL1-embedded WHA were performed on confluent cells for 24h and length traveled was evaluated using ImageJ. For the cell-spreading assay, (F) Cell lines were seeded for 6h on plastic and COL1 and cells showing an elongated morphology were counted manually. (G) Cell doubling time for Dt81Hepa1-6 and parental Hepa1-6 cells. (H) Viability of Dt81Hepa1-6 and Hepa1-6 cells following *in vitro* exposure to cisplatin [25ug/mL] for 24h. (*P<0.05; **P<0.01; ***P<0.001).

The aggregation of cells over a non-adherent surface assess their ability to grow and survive in absence of cell-cell contact [17]. We quantified the number of cells in aggregates when seeded on soft agar. The number of cells per aggregate was significantly higher in Hepa1-6 than Dt81Hepa1-6 (50.0±3.6 cells vs 7.0±0.9 cells; *P*<0.001) suggesting that Dt81Hepa1-6 required less cell-cell contact to survive and thus are more tumorigenic (Fig 4A). To further evaluate tumorigenicity *in vitro*, we used anchorage-independent growth and clonal colony formation assays [11]. Anchorage-independent growth measures individual cell growth in a non-adherent matrix of soft agar. When the ability to form colonies in soft agar was compared between Dt81Hepa1-6 cells and parental Hepa1-6 cells, the number of colonies was significantly higher with Dt81Hepa1-6 cells (12.3±3.3 colonies vs 34.7±6.8 colonies; *P*<0.05) (Fig 4B). Similarly, the number of colonies formed under the colony-forming assay was higher with Dt81Hepa1-6 cells than with parental Hepa1-6 cells (47.7±3.2 vs 1.3±0.9 colonies respectively; *P*<0.001). Since single isolated cells are used in the colony-forming assay, this result confirms the higher cell-cell independent growth ability of Dt81Hepa1-6 compared to parenteral Hepa1-6 cells (Fig 4C).

Invasion and motility are characteristics associated with metastatic potential and tissue invasiveness. The invasive potential of cells in a double-layer COL1 hemisphere is a surrogate marker for *in vivo* invasiveness [19]. Invasion of the outer COL1 layer was more important by parental Hepa1-6 than by Dt81Hepa1-6 cells (38412±6519 Area under the curve (AUC) vs. 13770±3184AUC; *P*<0.01) (Fig 4D). The ability of cells to move on a surface, or motility, is a complementary assay that evaluates the invasive potential of a cell line. In a wound-healing assay (WHA), Hepa1-6 cells showed higher mobility than Dt81Hepa1-6 on both plastic (1472±24AU vs. 423±49AU; *P*<0.001) and in COL1-embedded gel (1436±20AU vs. 505±28AU; *P*<0.001) (Fig 4E). Cell attachment, in opposition to anchorage-independent growth, allows the assessment of a cell line ability to adapt to an *in vitro* bidimensional environment [23]. The Hepa1-6 cells adopted a spread morphology significantly faster than Dt81Hepa1-6 cells 6h after seeding on plastic (19.2±1.2 cells vs. 0.2±0.2 cells; *P*<0.001) or on COL1 (74.7±1.6 cells vs. 27.3±0.6 cells; *P*<0.001) (Fig 4F). The cell doubling time of Dt81Hepa1-6 cells was also assessed and was significantly higher than that of Hepa1-6 cells (32.12±2.11 vs. 26.99±1.93 hours; *P*<0.001) (Fig 4G).

To assess the response of the Dt81Hepa1-6 cell line to antineoplastic compounds, viability of these cells was measured following *in vitro* cisplatin treatment [25ug/mL]. Viability of Dt81Hepa1-6 cells was significantly higher after treatment compared to Hepa1-6 cells (44.2±5.3% vs. 28.6±1,8%; *P*<0.01) (Fig 4H).

### Expression of EpCAM by Dt81Hepa1-6 cells

**Fig 5:**
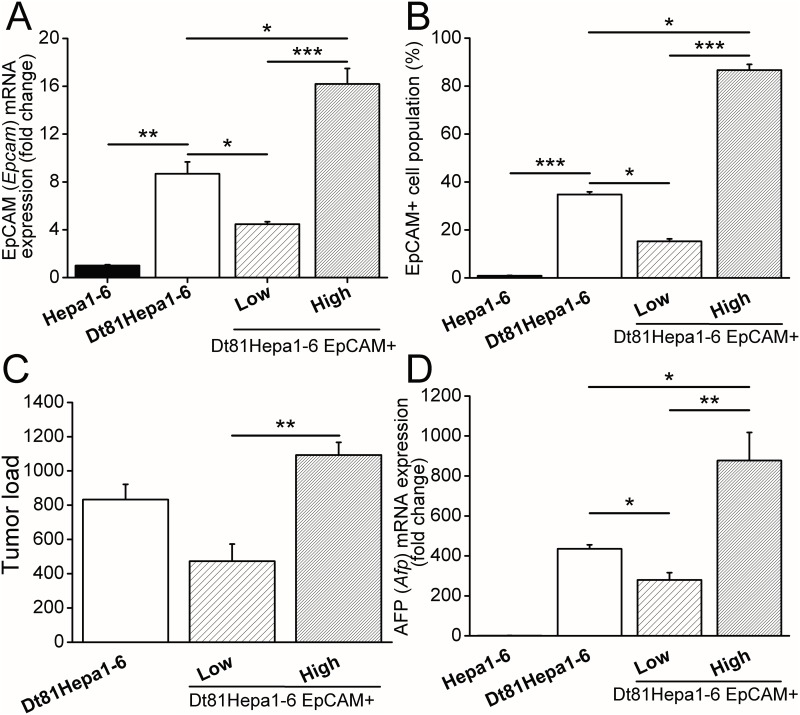
EpCAM+ Dt81Hepa1-6 cells are highly tumorigenic. Dt81Hepa1-6 cells were incubated with APC-tagged anti-CD326 (EpCAM) antibody and sorted by FACS as EpCAM+high and EpCAM+low Dt81Hepa1-6 cells. Hepa1-6, Dt81Hepa1-6, EpCAM+high and EpCAM+low were analyzed by (A) qPCR for EpCAM mRNA expression and (B) flow cytometry after labeling with APC-tagged anti-CD326 (EpCAM). Dt81Hepa1-6, EpCAM+low and EpCAM+high were injected intrasplenically in C57BL/6 mice and sacrificed after 21 days. (C) Tumor load (>0.5mm) was assessed and (D) *AFP* mRNA expression was compared to healthy liver controls. (*P<0.05; **P<0.01; ***P<0.001).

We next evaluated the expression profile of genes associated with poor tumor prognosis. *EpCAM* mRNA expression, a known marker of enhanced tumorigenicity and tumor initiating cells, was first evaluated. *EpCAM* mRNA was significantly upregulated in Dt81Hepa1-6 compared to Hepa1-6 (8.7±1.0 fold changes; *P*<0.01) (Fig 5A left). To confirm the presence of EpCAM at the surface of Dt81Hepa1-6 cells, we used flow cytometry and staining with anti-EpCAM fluorescent antibodies. The number of cells expressing EpCAM at their surface was significantly higher in Dt81Hepa1-6 than Hepa1-6 (34.8±1.1% vs. 0.9±0.1%; *P*<0.001) (Fig 5B left). To assess the role of EpCAM expression by Dt81Hepa1-6 cells, Dt81Hepa1-6 were sorted by flow cytometry based on their levels of EpCAM expression and cultured for 4 weeks in two groups: Dt81Hepa1-6 EpCAM+high and Dt81Hepa1-6 EpCAM+low. *In vitro* cultured Dt81Hepa1-6 EpCAM+high cells had approximately 4 fold higher expression level of EpCAM mRNA than Dt81Hepa1-6 EpCAM+low (Fig 5A right) and maintained high expression levels of EpCAM at the surface (86.7±2.3% EpCAM+ (Fig 5B right)).

To assess the contribution of EpCAM+ cells to Dt81Hepa1-6 tumerogenicity, Dt81Hepa1-6 EpCAM+high, Dt81Hepa1-6 EpCAM+low and non-sorted Dt81Hepa1-6 cells were injected intrasplenically (IS) in C57BL/6 mice. Dt81Hepa1-6 EpCAM+high cells were associated with higher tumor load than Dt81Hepa1-6 EpCAM+low cells (Tumor load (Fig 5C): 1093±74 tumors vs 473±100 tumors *P*<0.01) and AFP levels (Fig 5D: 877.3±140.7 fold changes vs. 279.6±36.4 fold changes; *P*<0.01). Unsorted Dt81Hepa1-6 cells resulted in tumor loads that were proportional to the levels of EpCAM positive cells within the unsorted population (Fig 5B and 5D).

### Tumor gene expression

**Fig 6:**
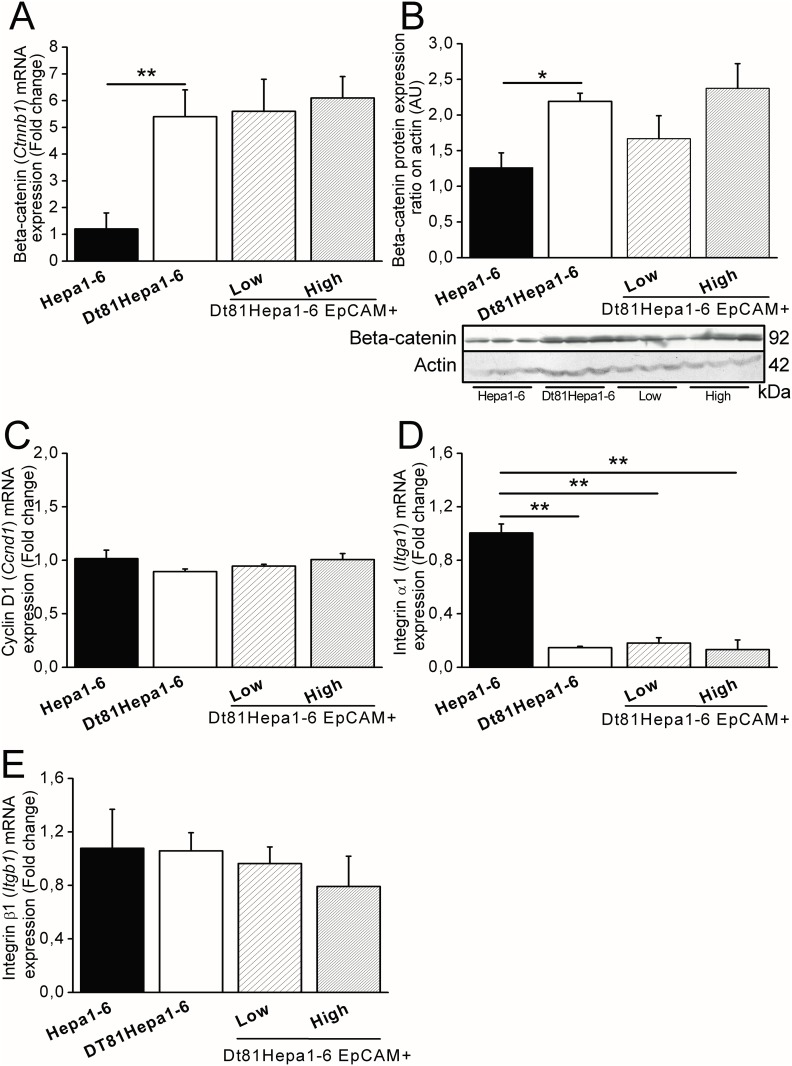
Expression of β-Catenin, Cyclin D1 and Integrins by Dt81Hepa1-6 cells. All experiments were performed on freshly trypsinized cells prior to their injection *in vivo*. Genes or proteins of interest were analyzed in Hepa1-6, Dt81Hepa1-6, EpCAM+low and EpCAM+high cell lines by qRT-PCR and/or western blot. (A) β-Catenin (*Ctnnb1)* gene and (B) protein expression shown on immunoblot (C) Cyclin D1 (Ccnd1), (D) Integrin-alpha1 (Itga1) and (E) Integrin-β1 (Itgb1) mRNA expression. (*P<0.05; **P<0.01).

The β-catenin (*Ctnnb1*) pathway regulates the expression of *EpCAM* [24]. The expression of β-catenin was measured at the mRNA and protein level. We found that Dt81Hepa1-6 cells expressed higher levels of β-catenin at the mRNA (Fig 6A: 5.4±1.0 fold changes in Hepa1-6: vs. 1.2±0.6 fold changes in Dt81Hepa1-6; *P*<0.01) and protein levels (Fig 6B). However, no significant differences in β-catenin expression level were found between Dt81Hepa1-6 EpCAM+ high and EpCAM+low cells (5.6±1.2 fold changes vs. 6.1±0.8 fold changes). Expression of Cyclin D1 (Ccnd1), a protein essential for cell growth and survival under control of EpCAM [25] was assayed. No significant differences were observed between mRNA levels of cyclinD1 in Dt81Hepa1-6 and Hepa1-6 cells or between EpCAM^-^ or EpCAM^+^ Dt81Hepa1-6 cells (Fig 6C).

In order to explain the difference in the rate of spreading between Dt81Hepa1-6 and parental Hep1-6 cells, the expression level of two attachment molecules was assessed. Integrin-alpha1 *(Itga1)* gene expression was lower in Dt81Hepa1-6 than parental Hepa1-6 (0.14±0.01 fold changes; *P*<0.01) (Fig 6D) while no changes were seen in integrin-β1 (Itgb1) expression (Fig 6E). No differences were observed in the expression level of integrins between Dt81Hepa1-6 EpCAM+high and EpCAM+low cells.

The expression level of pro-survival (AKT, ERK1/2 and Bcl-XL) and pro-apoptotic (Bak, Bad and Bid) proteins was assessed in Dt81Hepa1-6 cells and parental cells by western blotting. The expression level of these proteins was similar in Hepa1-6 and Dt81Hepa1-6 cells (S2 Fig).

## Discussion

Using *in vivo* passage of Hepa1-6 HCC cells in immunocompetent C57BL/6 mice, a different clone, Dt81Hepa1-6, with characteristics of tumor-initiating and stem/progenitor cells was isolated. Dt81Hepa1-6 cells are a homogenous cell population with a distinct morphology, different from that of parental Hepa1-6 cells. The cell line derived from this clone is very well adapted for *in vivo* growth in immunocompetent C57BL/6 mice showing a quasi-exponential time development curve characteristic of growth usually observed in cancer models. A cell dose of 1M cells resulted in the rapid and reproducible development of visible tumors. Furthermore, doses as low as 1000 cells were sufficient to trigger microscopic tumor development. A low-dose HCC model using these cells could be used to mimic the lengthy and gradual cancer development in immune competent hosts, allowing the testing of drugs designed to control HCC recurrence.

While these cells showed improved tumorigenicity compared to parental cells, Dt81Hepa1-6 cells maintained high liver-specificity as no tumors could be found in other organs. Only a few cells were observed at the site of injection when cells were injected intrasplenically, similar to parental Hepa1-6 intrasplenic cell injections. The ability to grow *in vitro* in an anchorage-independent manner is closely associated with tumorigenicity [26]. This mimics the process of tumor growth *in vivo*. Dt81Hepa1-6 cells showed increased anchorage-independent growth compared to Hepa1-6 parental cells. This ability could contribute to Dt81Hepa1-6 increased tumerogenicity *in vivo*. Besides anchorage-independence, we also tested the importance of cell-cell contacts in tumor growth as measured by the slow aggregation assay as described by Boterberg *et al*. [17]. This assay is useful to evaluate if cancer cells have decreased cell-cell contact interactions, a process often mediated by E-cadherin [17, 27]. Interestingly, E-cadherin expression can be abrogated by EpCAM [28], a cell surface molecule associated with highly tumorigenic cells [29]. The lower the number of cells per aggregates, the less those cells need cell-cell interaction to survive and proliferate [17]. The number of Dt81Hepa1-6 cells per aggregates was lower than Hepa1-6, suggesting that Dt81Hepa1-6 were indeed less dependent on cell-cell contact. The lower requirement of cell-cell contact by Dt81Hepa1-6 was confirmed by the colony formation assay, a factor also associated with tumorigenicity [11]. Our results show that isolated cells from the Dt81Hepa1-6 cell line led to the formation of proliferating colonies at a rate 50 times higher than parental Hepa1-6 cells. This is in accordance with *in vivo* experiments showing the ability of Dt81Hepa1-6 cells to rapidly form visible tumors compared to Hepa1-6 cells.

Cell spreading, a step of the cell attachment process, was more rapid in Hepa1-6 than Dt81Hepa1-6 cells on both plastic and COL1 matrix. This could be explained by the lower expression of integrin-alpha1 found in Dt81Hepa1-6; integrin-alpha1 is known to interact with COL1 when paired with integrin-β1 [30]. This result and their higher cell doubling time, which may seem contradictory to our *in vivo* results, may in fact reflect the lessened ability of Dt81Hepa1-6 to form monolayers, a well-known hallmark of TIC [12, 31].

*In vitro* motility, invasiveness and rapid cell division are associated with metastatic potential and tissue invasion *in vivo*. Unexpectedly, Dt81Hepa1-6 cells showed lower *in vitro* motility and invasiveness and higher cell doubling time. Studies by Li *et al*. have shown that by selecting metastasis of a certain tumor type, one was able to modulate the metastatic potential of cancer cells [32]. In the present study, cells were selected for their ability to form tumors in the liver as intrasplenical injection provides a diffuse portocentric implantation method [33]. Our subcutaneous experiment confirms the adaptation of Dt81Hepa1-6 cells for growth inside the liver and not in other organs. Interestingly, EpCAM overexpression in many different cell line models such as lung, ovarian, colon and bladder cancer has been associated with low invasiveness potential [34, 35].

Expression of EpCAM by tumor cells is associated with increased tumorigenicity and anchorage-independent growth [36]. The exact mechanisms by which EpCAM exert its role remains unclear, but it is currently believed that homologous activation would occur through other EpCAM molecules through cell-cell contact [36]. Activation of EpCAM would result in the cleavage of its intracellular domain (EpCID) that would then be co-translocated with β-catenin to the nucleus leading to the activation of several genes associated with survival, proliferation and pluripotentiality such as *nanog*, *sox2*, *cmyc* and *cyclin D1* [36]. Assessment of EpCAM expression at the cell surface by flow cytometry showed that a large subset of Dt81Hepa1-6 cells expressed EpCAM while few or no EpCAM positive cells were found among Hepa1-6 cells. This result suggests that Dt81Hepa1-6 cells, as suggested by the TIC model [37], are a heterogeneous cell population with a cell subset having an improved ability to form tumors *in vivo*. Interestingly, the well-characterized and widely used HCC cell line, HuH7, displays similar heterogeneity in EpCAM expression level [13, 24]. This validates EpCAM as a marker of tumorigenicity in HCC cells, as cells selected for their expression of EpCAM showed an increases ability to form tumors *in vivo*. It also confirms the importance of EpCAM-positive cell subpopulations in HCC progression [36]. In addition to EpCAM, Dt81Hepa1-6 cells express both CD24 and CD44 (data not shown), two markers of increased tumorigenicity that have also been associated with cancer stem cells [38, 39].

Overexpression of β-catenin, an important membrane-to-nucleus messenger molecule, has been shown to be associated with HCC aggressiveness [40]. β-catenin expression can lead to the induction of EpCAM transcription and, in association with EpCAM, allows it to play its role as a transcriptional regulator [41]. Expression of β-catenin was increased over 6 fold in Dt81Hepa1-6 compared to parental cells. However, mRNA and protein expression of β-catenin did not differ between EpCAM+low and EpCAM+high cells suggesting that while this pathway is enhanced in Dt81Hepa1-6, it may not be the main regulator of EpCAM expression at the cell surface. Therefore, we can safely conclude from our data that while β-catenin is found at higher levels in Dt81Hepa1-6 and likely potentiates EpCAM effect on tumorigenicity, it is not directly responsible for the higher levels of EpCAM found within the EpCAM+ Dt81Hepa1-6 subset.

Interestingly, Dt81Hepa1-6 cells showed increased viability following exposure to antineoplastic cisplatin compared to Hepa1-6 cells. This suggests that Dt81Hepa1-6 cells could be more resistant to the proapoptotic effect of cisplatin than Hepa1-6 cells [42, 43]. However, no differences in the levels of pro-apoptotic Bak, Bad and Bid and pro-survival AKT, ERK1/2 and Bcl-XL were observed between Dt81Hepa1-6 and Hepa1-6 cells. These observations suggest that other modifications could be involved since cisplatin resistance can occur through several molecular pathways [42, 43]. Further research is needed to identify the factor(s) responsible for this increased resistance to cisplatin by Dt81Hepa1-6 cells.

In conclusion, Dt81Hepa1-6 cells, isolated from a tumor selected through an *in vivo* passage, show a drastically increased tumorigenicity while maintaining high specificity for the liver. This phenotype is associated with an increased expression of EpCAM and β-catenin by these cells. Dt81Hepa1-6 cells are characterized by the presence of a large population of EpCAM+ cells, a hallmark of tumor inducing cells. These cells show all the characteristics of a hepatocellular carcinoma cell line with tumor-initiating characteristics. The cancer stem cell-like properties of Dt81Hepa1-6 will make this cell line a useful tool for the *in vivo* study of hepatocellular carcinoma in immunocompetent hosts.

## Supporting information

S1 Fig Isolation of the Dt81Hepa1-6 cell line. A suspension of 1M Hepa1-6 cells was injected intrasplenically in C57BL/6 mice (n = 30). (A) HPS-stained liver slices 21 days post-inoculation and (B) whole liver photographs 70 days post-inoculation. (C) Expression of albumin (Alb) was assessed and compared to Hepa1-6 and normal primary mouse hepatocytes to confirm the hepatic origin of Dt81Hepa1-6. (D) Morphology of Dt81Hepa1-6 and parental Hepa1-6 cells in culture.
(TIFF)

S2 Fig Expression of pro-survival AKT, ERK1/2 and Bcl-XL and pro-apoptotic Bak, Bad and Bid by Dt81Hepa1-6 and parental cells. Protein was evaluated in both cell lines after 24h in culture with non-supplemented DMEM. Cells were at 70% confluence and seeded on COL1 [13.9μg/cm^2^]. Immunoblots were obtained of pro-survival proteins: AKT, ERK1/2 and Bcl-XL; and pro-apoptotic proteins: Bak, Bad and Bid.
(TIFF)
