# Supplementary figures and images for: Highly tumorigenic hepatocellular carcinoma cell line with cancer stem cell-like properties
Source: PLoS One. 2017 Feb 2;12(2):e0171215. doi: 10.1371/journal.pone.0171215 (PMC5289561; doi:10.1371/journal.pone.0171215)

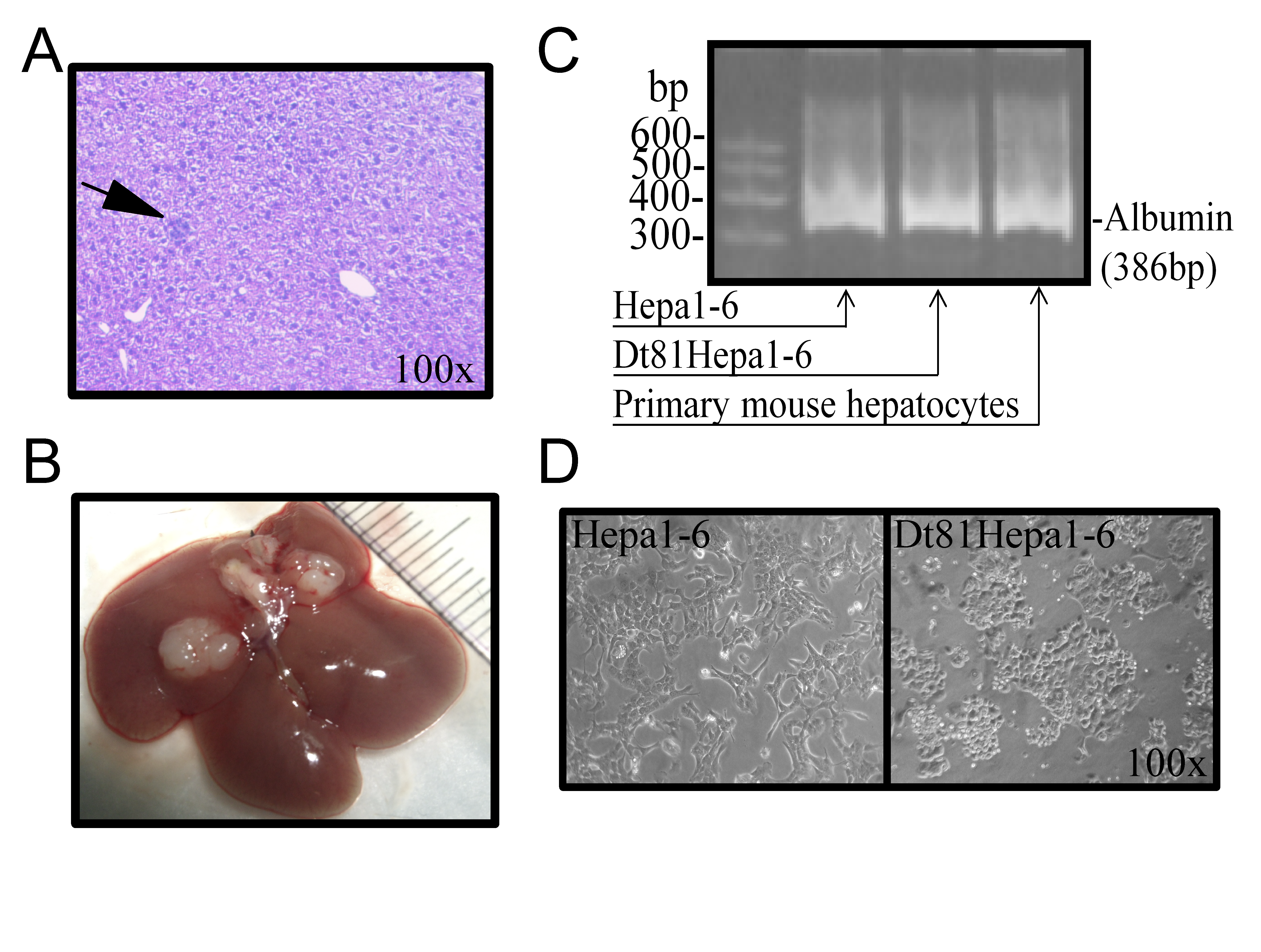

Supplement: S1 Fig — A suspension of 1M Hepa1-6 cells was injected intrasplenically in C57BL/6 mice (n = 30). (A) HPS-stained liver slices 21 days post-inoculation and (B) whole liver photographs 70 days post-inoculation. (C) Expression of albumin (Alb) was assessed and compared to Hepa1-6 and normal primary mouse hepatocytes to confirm the hepatic origin of Dt81Hepa1-6. (D) Morphology of Dt81Hepa1-6 and parental Hepa1-6 cells in culture. (TIFF) [file pone.0171215.s001.tiff]

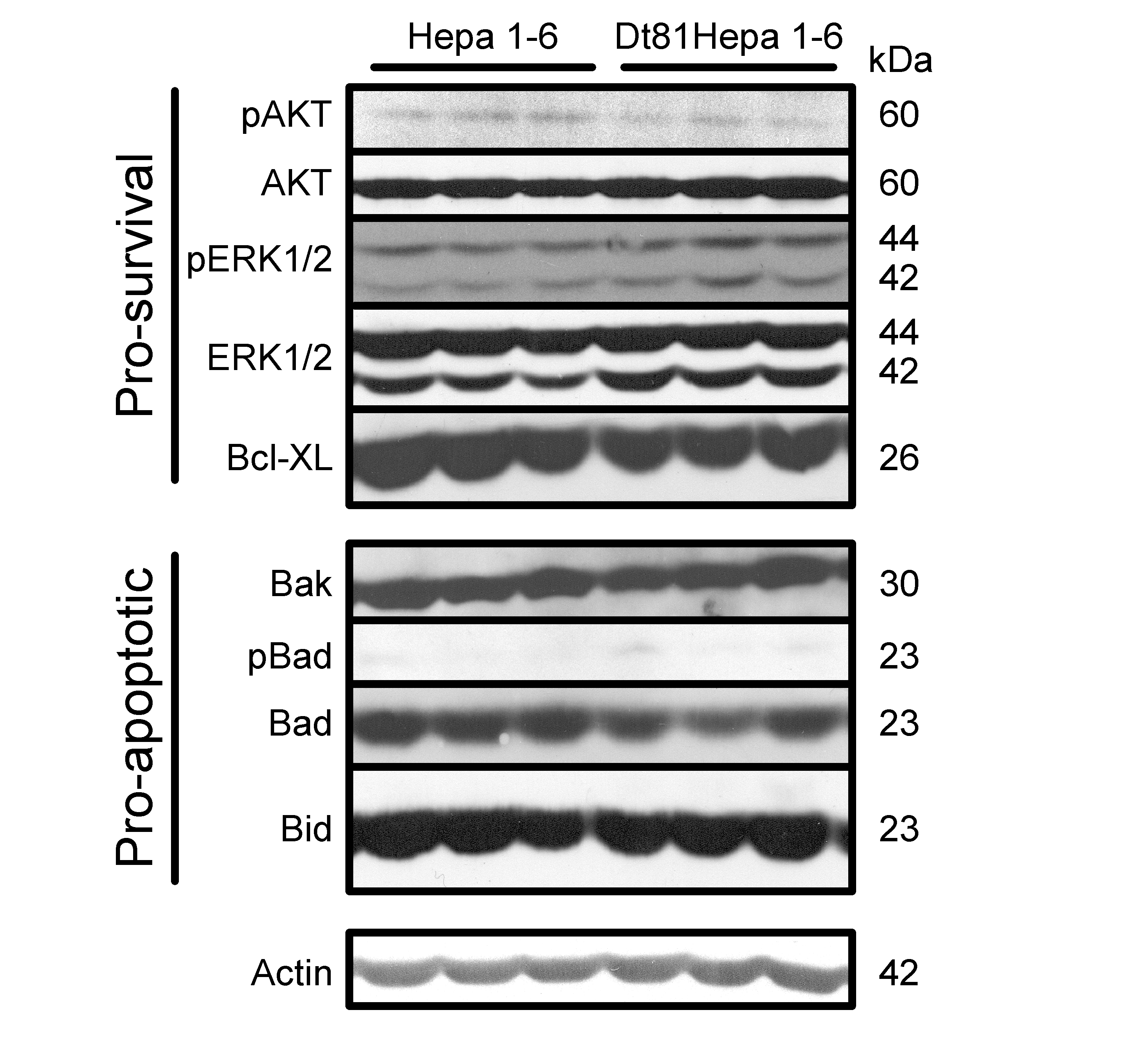

Supplement: S2 Fig — Protein was evaluated in both cell lines after 24h in culture with non-supplemented DMEM. Cells were at 70% confluence and seeded on COL1 [13.9μg/cm2]. Immunoblots were obtained of pro-survival proteins: AKT, ERK1/2 and Bcl-XL; and pro-apoptotic proteins: Bak, Bad and Bid. (TIFF) [file pone.0171215.s002.tiff]
